# A TetR family transcriptional regulator, SP_2854 can affect the butenyl-spinosyn biosynthesis by regulating glucose metabolism in *Saccharopolyspora pogona*

**DOI:** 10.1186/s12934-022-01808-2

**Published:** 2022-05-14

**Authors:** Jie Rang, Ziyuan Xia, Ling Shuai, Li Cao, Yang Liu, Xiaomin Li, Jiao Xie, Yunlong Li, Shengbiao Hu, Qingji Xie, Liqiu Xia

**Affiliations:** 1 Hunan Provincial Key Laboratory for Microbial Molecular Biology, State Key Laboratory of Development Biology of Freshwater Fish, College of Life Science, Hunan Normal University, Changsha, 410081 China; 2 Key Laboratory of Chemical Biology and Traditional Chinese Medicine Research (MOE of China), National & Local Joint Engineering Laboratory for New Petro-Chemical Materials and Fine Utilization of Resources, College of Chemistry and Chemical Engineering, Hunan Normal University, Changsha, 410081 China

**Keywords:** *Saccharopolyspora pogona*, Butenyl-spinosyn, SP_2854, Omics analysis, Metabolic engineering

## Abstract

**Background:**

Butenyl-spinosyn produced by *Saccharopolyspora pogona* exhibits strong insecticidal activity and a broad pesticidal spectrum. Currently, important functional genes involve in butenyl-spinosyn biosynthesis remain unknown, which leads to difficulty in efficiently understanding its regulatory mechanism, and improving its production by metabolic engineering.

**Results:**

Here, we identified a TetR family transcriptional regulator, SP_2854, that can positively regulate butenyl-spinosyn biosynthesis and affect strain growth, glucose consumption, and mycelial morphology in *S. pogona*. Using targeted metabolomic analyses, we found that SP_2854 overexpression enhanced glucose metabolism, while SP_2854 deletion had the opposite effect. To decipher the overproduction mechanism in detail, comparative proteomic analysis was carried out in the SP-2854 overexpressing mutant and the original strain, and we found that SP_2854 overexpression promoted the expression of proteins involved in glucose metabolism.

**Conclusion:**

Our findings suggest that SP_2854 can affect strain growth and development and butenyl-spinosyn biosynthesis in *S. pogona* by controlling glucose metabolism. The strategy reported here will be valuable in paving the way for genetic engineering of regulatory elements in actinomycetes to improve important natural products production.

**Supplementary Information:**

The online version contains supplementary material available at 10.1186/s12934-022-01808-2.

## Background

Butenyl-spinosyn is produced by *Saccharopolyspora pogona* (*S. pogona*) originally isolated from soil samples collected in Indiana, U.S.A., in 1990 [1, 2]. It is structurally and chemically related to spinosyn produced by *Saccharopolyspora spinosa* [2, 3]. Butenyl-spinosyn has excellent activity against various insects, such as Lepidoptera, Diptera, and Coleoptera, but has little or no effect on a broad range of nontarget insects and mammals, making it one of the most promising biological pesticides [4, 5]. At present, the butenyl-spinosyn biosynthetic gene cluster (*bus* cluster) and its biosynthetic pathway have been elaborated [2, 6]. Although much progress has been made in understanding the butenyl-spinosyn biosynthetic pathway, little is known about the regulation of butenyl-spinosyn biosynthesis. To date, only a few regulators have been identified as being involved in the regulation of butenyl-spinosyn production in *S. pogona*, including the SenX3/RegX3 system, Sp1418, Sp13016, SP_1288 and an AfsR regulatory factor [7–11]. A better understanding of the regulatory mechanisms of butenyl-spinosyn biosynthesis will provide important insights for strain improvement by metabolic engineering.

A number of regulatory genes with pleiotropic effects on the biosynthesis of natural products in actinomycetes have been identified and studied extensively [12–14]. Broadly speaking, we can divide the regulatory genes that control natural product biosynthesis into two types. First, some genes are located in natural product biosynthetic gene clusters together with genes for biosynthesis and secretion of natural products and are usually pathway-specific [15]. For example, the regulator genes *actII-orf4*, *cdaR* and *redD* exist in the *Streptomyces coelicolor* genome, and their coding proteins control actinorhodin, calcium-dependent antibiotic (CDA), and undecylprodigiosin biosynthesis, respectively [16, 17]. Second, other genes are global regulators that control multiple metabolic pathways and may not be linked to specific biosynthetic gene clusters [18]. For example, the two-component PhoR-PhoP system is well studied for the regulation of antibiotic biosynthesis induced by phosphate starvation [15]. TetR family regulatory factors (TFRs), a class of regulators commonly found in actinomycetes, participate in diverse cellular processes [19, 20]. In recent years, a variety of TFRs, as pathway-specific or global regulators, were identified as regulating the biosynthesis of natural products by binding to the promoter region of their target genes [21]. Whole genome sequencing results showed that at least 86 putative TFRs were encoded in the genome of *S. pogona* (CP031142). Therefore, the TFRs associated with butenyl-spinosyn production in *S. pogona* need to be identified to improve our understanding of the regulatory mechanism underlying butenyl-spinosyn biosynthesis.

The biosynthetic regulatory network of actinomycete natural products is robust and tightly regulated. Although previous studies have showed that metabolic engineering of key regulatory factors advances our understanding of natural product biosynthesis [22, 23], omics techniques are required to dissect detailed regulatory mechanisms that may reveal the natural product biosynthesis regulatory network [24–26]. Recently, proteomics has become an efficient and cost-effective strategy for dissecting the biological functions of specific regulatory factors [27]. For example, the regulatory effect of the GlnR-mediated response to nitrogen limitation in *Streptomyces coelicolor* M145 was described in detail following proteomic analysis of a wild-type and a mutant strain [28]. In addition to proteomics, metabolomics has also been widely used to reveal the role of regulatory factors in natural product biosynthesis by depicting intracellular metabolic profiles [29]. For example, introduction of *adpA* into *Streptomyces* ZYJ-6 significantly improved candicidin productivity to achieve a concentration of 9338 μg/mL. Combined metabolic flux and metabolomics analyses indicated that methylmalonyl-CoA played a central role in candicidin production and that the *methB* gene responsible for the biosynthesis of methylmalonyl-CoA might be a candidate gene target for the AdpA regulatory factor [30]. However, few studies have used proteomic and metabolomic analyses to reveal the regulatory mechanisms of TFRs in natural product biosynthesis.

Here, we identified a TFR, SP_2854, that positively regulates butenyl-spinosyn biosynthesis. A comparative proteomic and metabolomic approach confirmed that SP_2854, as the global regulatory factor, affects butenyl-spinosyn biosynthesis by regulating glucose metabolism in addition to affecting the transcription of the *bus* cluster. This work deepens our understanding of the butenyl-spinosyn regulatory mechanism and lays an important foundation for studying the regulatory mechanism of SP_2854 in natural product biosynthesis in other actinomycetes.

## Results

### Construction of SP_2854 deletion and overexpression mutants

**Fig. 1:**
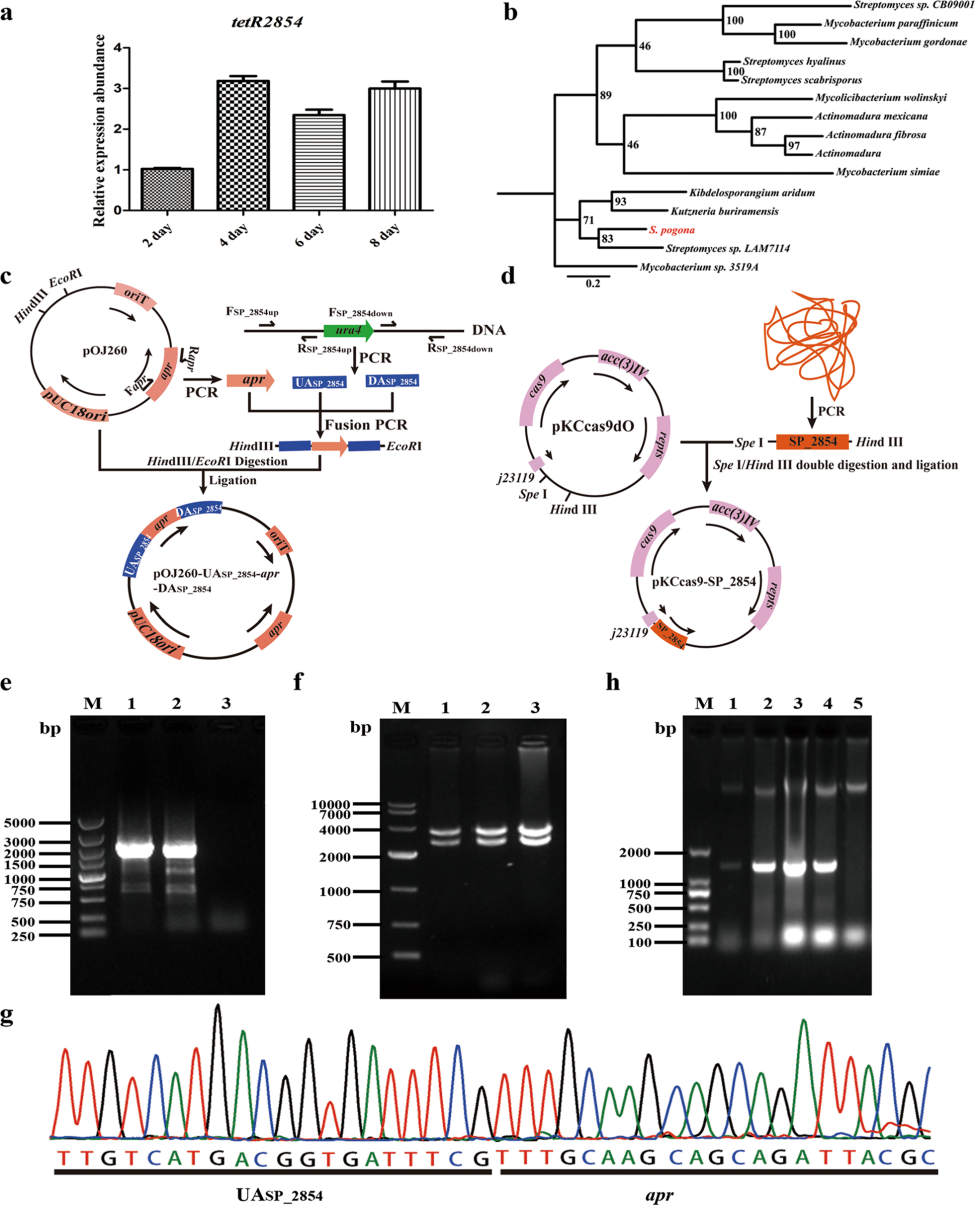
Deletion and overexpression of SP_2854. **A** Transcription levels of SP_2854 at different time points via qRT–PCR analysis. **B** Neighbour-joining (NJ) distance tree constructed using the amino acid sequence SP_2854 from actinomycetes using MEGA6. **C** Schematic diagrams of the *S. pogona*-ΔSP_2854 mutant. **D** Schematic diagrams of the *S. pogona*::SP_2854 mutant. **E** PCR identification of the *S. pogona*-ΔSP_2854 mutant. Wild-type *S. pogona* genomic DNA was used as the control. Lane M, DL5000 DNA marker; Lanes 1 and 2, *S. pogona*-ΔSP_2854 mutant; Lane 3, control. The tested transformant showed a 2.3-kb PCR amplicon, whereas the control showed no band in that location. **F** PCR identification of the *S. pogona*::SP_2854 mutant. Wild-type *S. pogona* genomic DNA was used as the control. Lane M, DL2000 DNA marker; Lanes 1–4, *S. pogona*::SP_2854 mutant; Lane 5, control. The tested transformant showed a 1.3-kb PCR amplicon, whereas the control showed no band that location. **G** Sequencing of the *S. pogona*-ΔSP_2854 mutant PCR amplicon

Whole genome sequencing results showed that at least 86 putative TFRs were encoded in the genome of *S. pogona*. Combined with previous comparative transcriptomic data mining studies, a new transcriptional regulator SP_2854, which was differentially expressed the strain growth logarithmic phase, attracted our attention. Therefore, SP_2854 was selected as a modified target to investigate whether it regulates primary metabolism and butenyl-spinosyn biosynthesis. According to the available genome sequence of *S. pogona*, SP_2854 consists of 213 amino acids with a molecular mass of approximately 24 kDa. qRT–PCR results showed that SP_2854 could be transcribed throughout the *S. pogona* growth cycle (Fig. 1a). Blast analysis revealed that SP_2854 homologues were widely distributed among actinomycetes, suggesting that this gene may have important biological functions in actinomycetes (Fig. 1b).

To define the role of SP_2854 in butenyl-spinosyn biosynthesis, plasmids pOJ260 and pKCcas9dO were selected as the editing tools for SP_2854 deletion and overexpression, respectively [31, 32]. To generate the SP_2854 deletion mutant, the recombinant plasmid pOJ260-UA_SP_2854_-*apr*-DA_SP_2854_ was constructed and then transferred into *S. pogona* through protoplast transformation (Fig. 1c). Since pOJ-260 is a suicide plasmid, it cannot be replicated in *S. pogona* and will eventually be lost as the strain grows. The resulting mutant, named *S. pogona*-ΔSP_2854, was verified by PCR with the primers F_SP_2854up_/R_*apr*_ for multiple independent transformants, and a 2.3-kb band was observed; no band was observed for the original strain of *S. pogona* (Fig. 1e). The results of *Hin*d III/*Eco*R I double digestion were also in line with expectations (Fig. 1f). Sequencing of the PCR amplicons showed that the 668-bp DNA region was completely deleted (Fig. 1g). For SP_2854 overexpression, the recombinant plasmid pKCcas9-SP_2854 was constructed and then transferred into *S. pogona* through protoplast transformation (Fig. 1d). Since pKCcas9dO can be replicated in *S. pogona*, it was only necessary to verify the presence of the *apr* gene in transformants. The resulting mutant, named *S. pogona*::SP_2854, was verified by PCR with the primers F_*apr*_/R_*apr*_ for multiple independent transformants, and a 1.3-kb band was observed; no band was observed for the original strain of *S. pogona* (Fig. 1h). qRT–PCR analysis revealed that the transcriptional level of SP_2854 in the *S. pogona*::SP_2854 mutant was significantly higher than that of the original strain, but no SP_2854 transcripts were detected in the mutant *S. pogona*-ΔSP_2854.

### SP_2854 plays a positive regulatory role in the butenyl-spinosyn biosynthesis

**Fig. 2:**
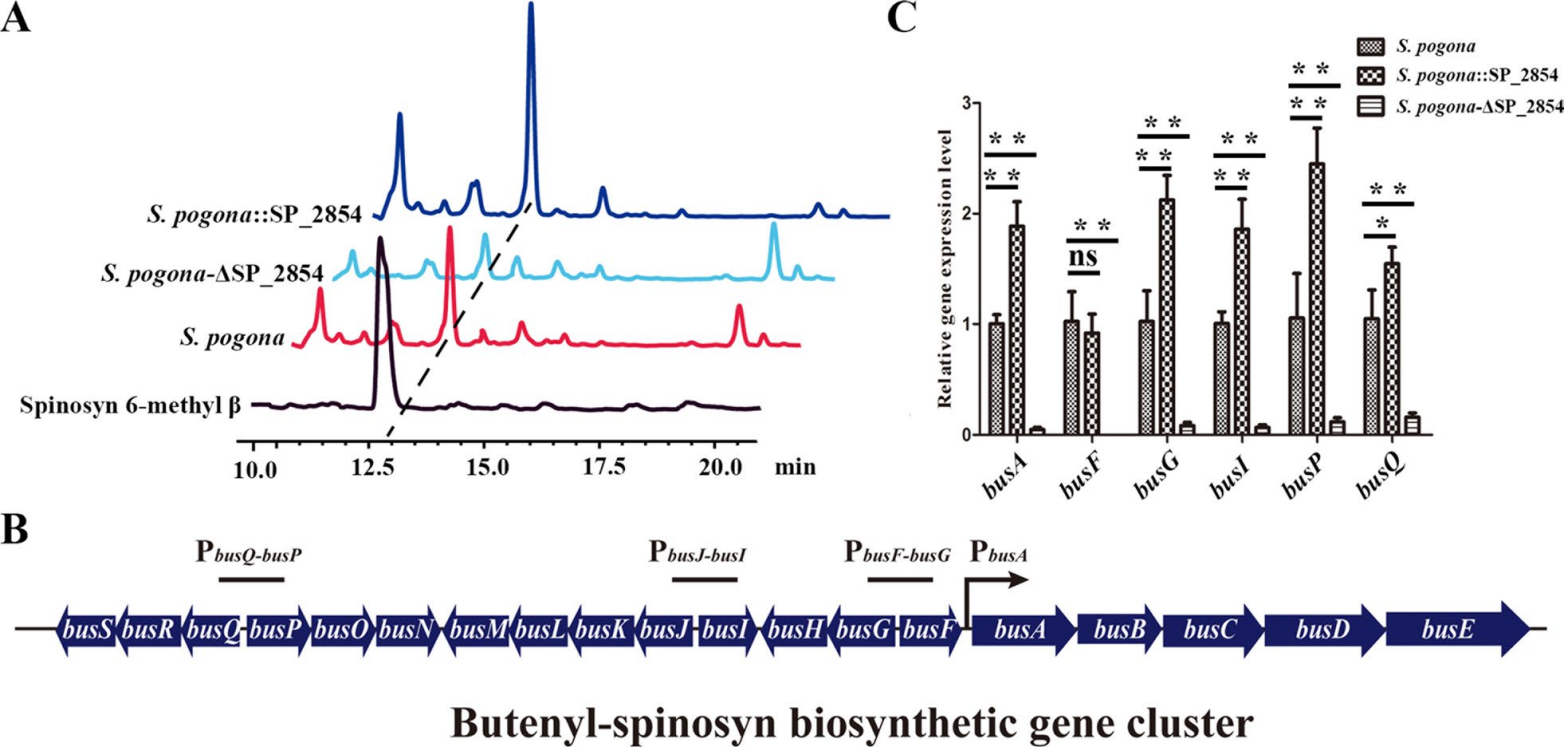
SP_2854 positively regulates butenyl-spinosyn production in *S. pogona*. **A** Comparison of butenyl-spinosyn production in wild-type *S. pogona* and the *S. pogona*-ΔSP_2854 and *S. pogona*::SP_2854 mutants via HPLC analysis. Eight-day fermentation liquid was pretreated for HPLC analysis. **B** Genetic organization of the *bus* cluster in *S. pogona*. **C** Effects of SP_2854 deletion and overexpression on the transcription level of the *bus* cluster. The cells of the different strains were inoculated into SFM and cultured at 30 °C for 4 days. Total RNA was then isolated and used for qRT–PCR assays. The control strain was wild-type *S. pogona*. 16S rRNA served as the normalization control

To identify the effects of SP_2854 deletion and overexpression on butenyl-spinosyn biosynthesis, we estimated the production of the *S. pogona*-ΔSP_2854 and *S. pogona*::SP_2854 mutants by comparing their butenyl-spinosyn chromatographic peak areas with that of the original strain under the same conditions. Previous studies confirmed that the chromatographic peak at 13 min was the butenyl-spinosyn component spinosyn 6-methyl β [9]. Therefore, the peak at 13 min was collected and identified by LC–MS/MS, and the result showed that (M + H) + ions at m/z = 633.17 contained a rhamnose ion fragment of 189 molecular mass (Additional file 1: Fig. S1), which is consistent with previous data [9]. Next, the chromatographic peak at 13 min was used as a basis for comparing changes in butenyl-spinosyn production in different strains. The results showed that overexpression of SP_2854 significantly promoted butenyl-spinosyn biosynthesis, and its yield reached 65.12 ± 8.57 mg/L, which was obviously higher than that of the original strain (22.48 ± 4.53 mg/L). Only a small amount of butenyl-spinosyn was produced in *S. pogona*-∆SP_2854 (5.10 ± 0.32 mg/L) (Fig. 2a). To determine the effect of SP_2854 on butenyl-spinosyn biosynthesis in detail, the expression levels of the *bus* cluster in the mutants and the original strain were studied by qRT–PCR (Fig. 2b). We found that SP_2854 deletion resulted in a significant decrease in the expression of *busA*, *busF*, *busG*, *busI*, *busP* and *busQ*, but, except for *busF*, SP_2854 overexpression promoted their expression, indicating that SP_2854 had a positive effect on butenyl-spinosyn biosynthesis (Fig. 2c).

### SP_2854 regulates strain growth and development, glucose consumption and mycelial morphology but not sporulation

**Fig. 3:**
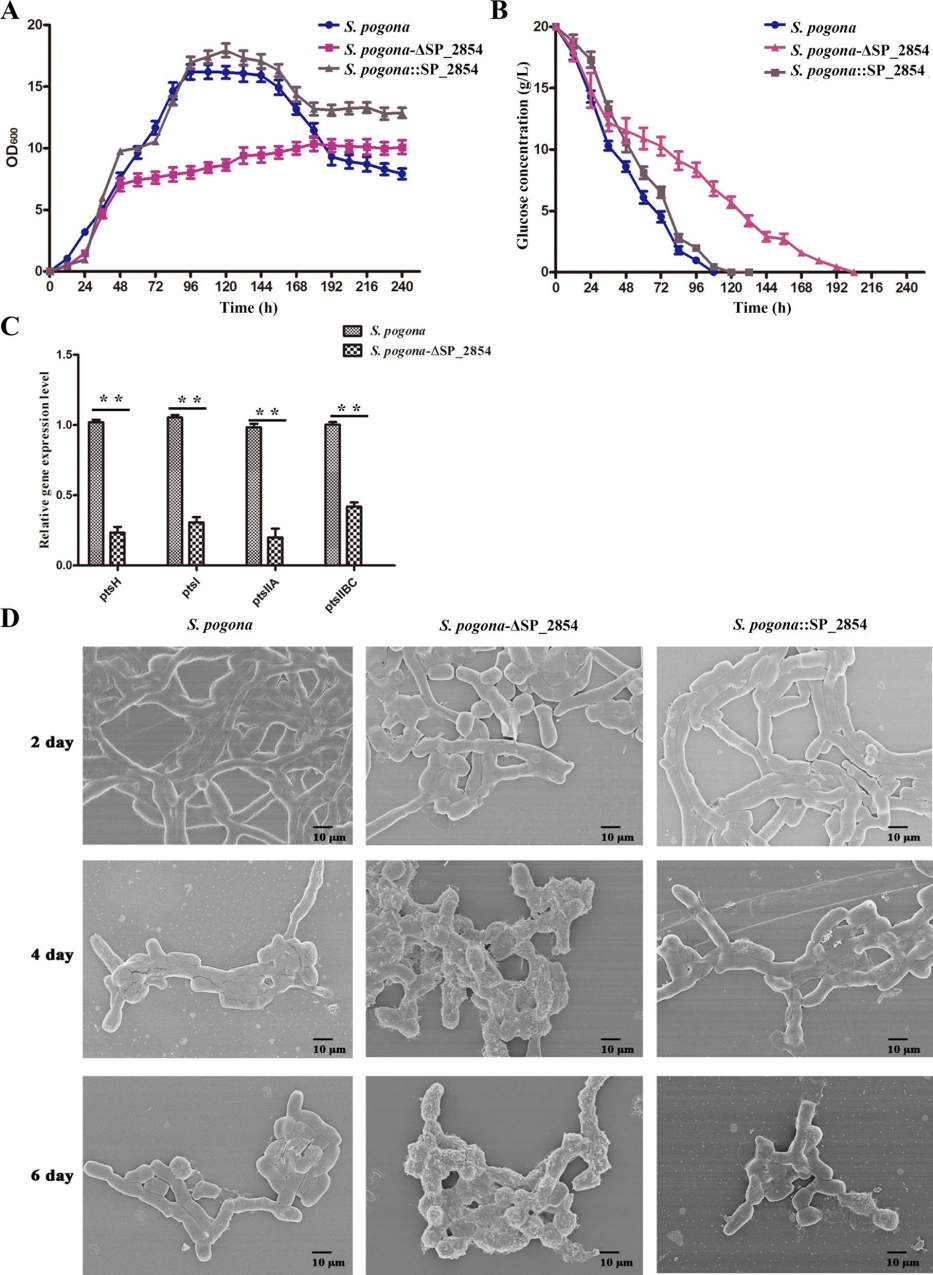
Effects of SP_2854 deletion and overexpression on strain growth, glucose consumption and mycelial morphology. **A** Growth curve analysis of wild-type *S. pogona* and the *S. pogona*-ΔSP_2854 and *S. pogona*::SP_2854 mutants in SFM. **B** Comparison of glucose utilization in wild-type *S. pogona* and the *S. pogona*-ΔSP_2854 and *S. pogona*::SP_2854 mutants in SFM. **C** Effects of SP_2854 deletion on the transcription levels of PTS^Glc^ genes involved in glucose uptake. The cells of the different strains were inoculated into SFM and cultured at 30 °C for 4 days. Total RNA was then isolated and used for qRT–PCR assays. The control strain was wild-type *S. pogona*. 16S rRNA served as the normalization control. **D** Scanning electron micrographs of wild-type *S. pogona* and the *S. pogona*-ΔSP_2854 and *S. pogona*::SP_2854 mutants. Comparison of mycelial morphology between the mutants and wild-type *S. pogona* on the 2nd, 4th and 6th days

TFRs often play much broader roles, such as in growth and development, nutrition, metabolism, and morphological differentiation [14], so we compared the mutants and the original strain in growth density, glucose consumption, mycelial morphology and sporulation. The results showed that the *S. pogona*::SP_2854 mutant and the original strain had similar growth rates in early growth, but bacterial density in the stationary phase and decline phase was higher in the mutant than in the original strain. The *S. pogona*-ΔSP_2854 mutant, however, had a significantly lower growth rate than that of the original strain (Fig. 3a). Glucose consumption analysis showed that the changing trend was consistent with growth density, which was reduced during the logarithmic phase and was almost exhausted late in the stationary phase. We found that the *S. pogona*::SP_2854 mutant and the original strain had similar glucose consumption rates; however, the *S. pogona*-ΔSP_2854 mutant required more time to complete glucose consumption in the environment than the original strain (8.5 days vs. 5 days) (Fig. 3b). To explain the effect of SP_2854 deletion on glucose uptake in strain, we performed qRT–PCR analysis on mutant *S. pogona*-ΔSP_2854 and the original strain to detect the mRNA levels for *ptsH*, *ptsI*, *ptsIIA* and *ptsIIBC* (phosphoenolpyruvate-dependent glucose phosphotransferase system genes, PTS^Glc^), which are important genes involved in glucose transport. The result showed that SP_2854 deletion resulted in a significant decrease in the expression of these genes (Fig. 3c). Scanning Electronic Microscopy (SEM) observation showed that there was no significant difference between *S. pogona*::SP_2854 and the original strain in mycelial morphology. However, some mycelia of the *S. pogona*-ΔSP_2854 mutant presented short rods or ellipsoid shapes in early growth (2 days) and exhibited obvious protrusions on the surface of mycelia at the stationary phase (4 days and 6 days) (Fig. 3d). With regard to sporulation, the mutants and the original strain had no obvious difference (Additional file 1: Fig. S2). These results indicate that SP_2854 can regulate strain growth, glucose consumption and mycelial morphology but is not involved in sporulation.

### SP_2854 can enhance acetyl-CoA supply to promote butenyl-spinosyn biosynthesis

**Fig. 4:**
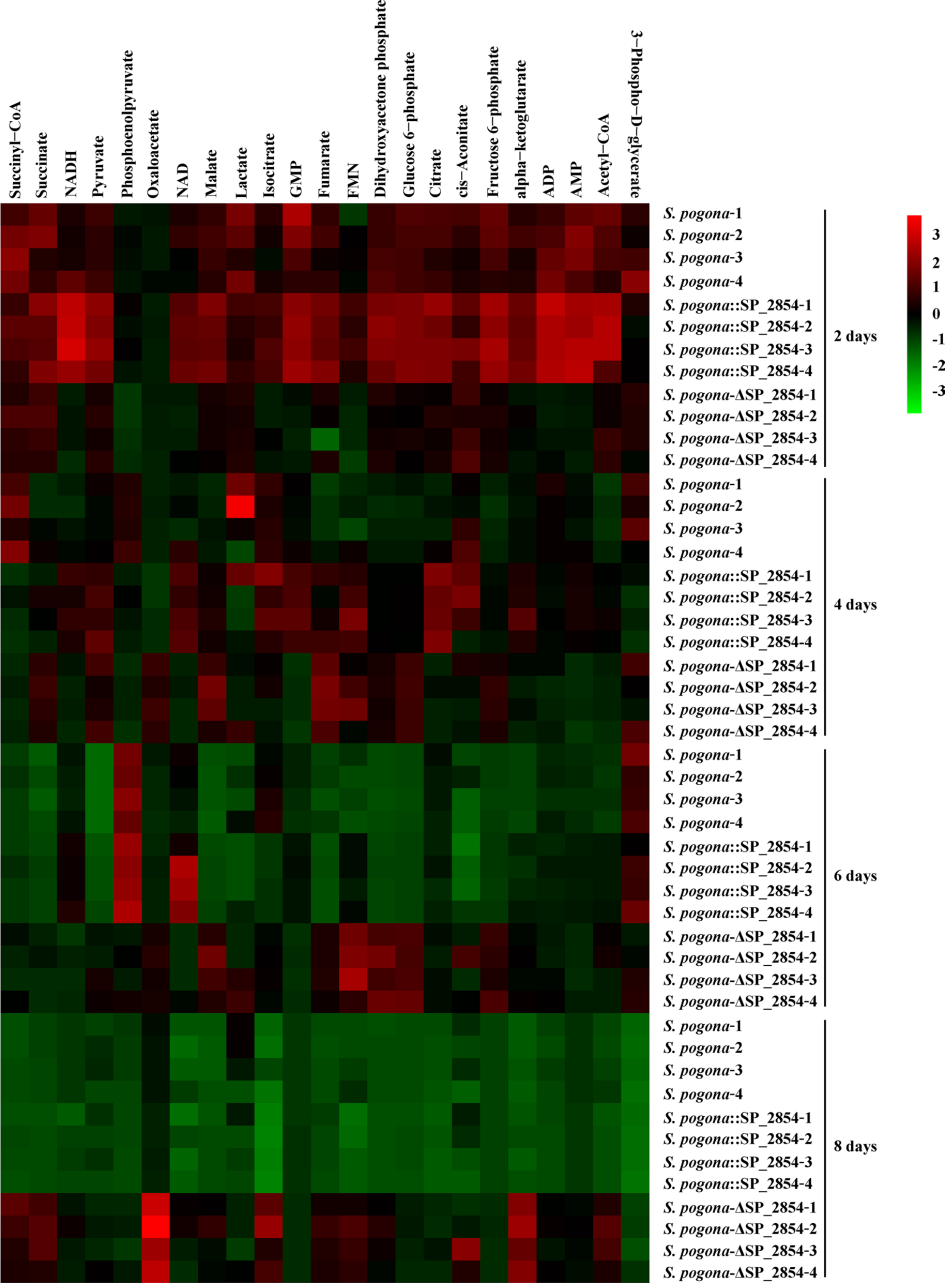
Heatmap with hierarchical clustering of all samples. Different colours represent distinct relative abundances of metabolites

**Fig. 5:**
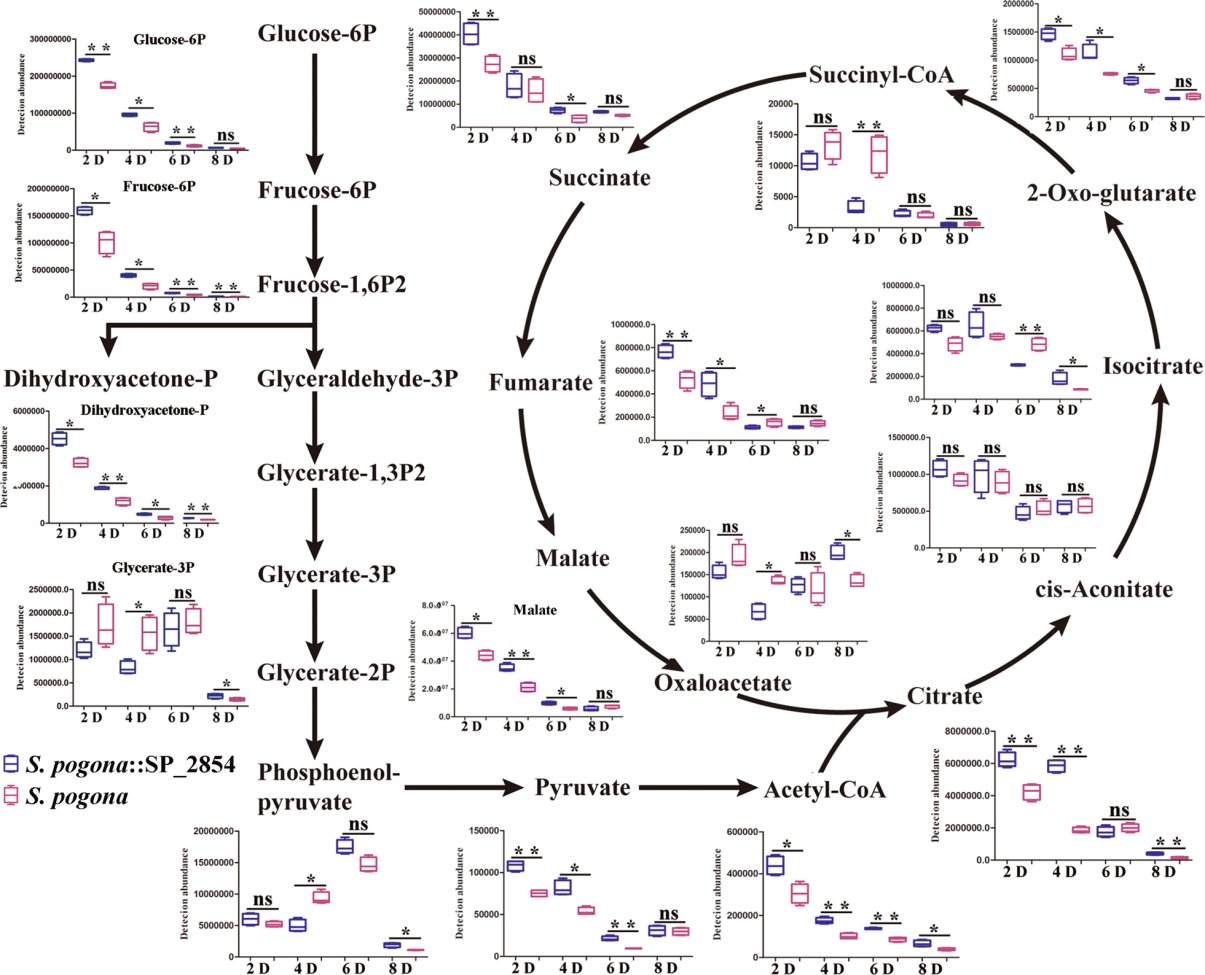
Comparison of the abundance of intracellular metabolites in the *S. pogona*::SP_2854 mutant (purple) and wild-type *S. pogona* (pink) related to the glycolysis pathway and TCA cycle (mean values from four biological replicates)

**Fig. 6:**
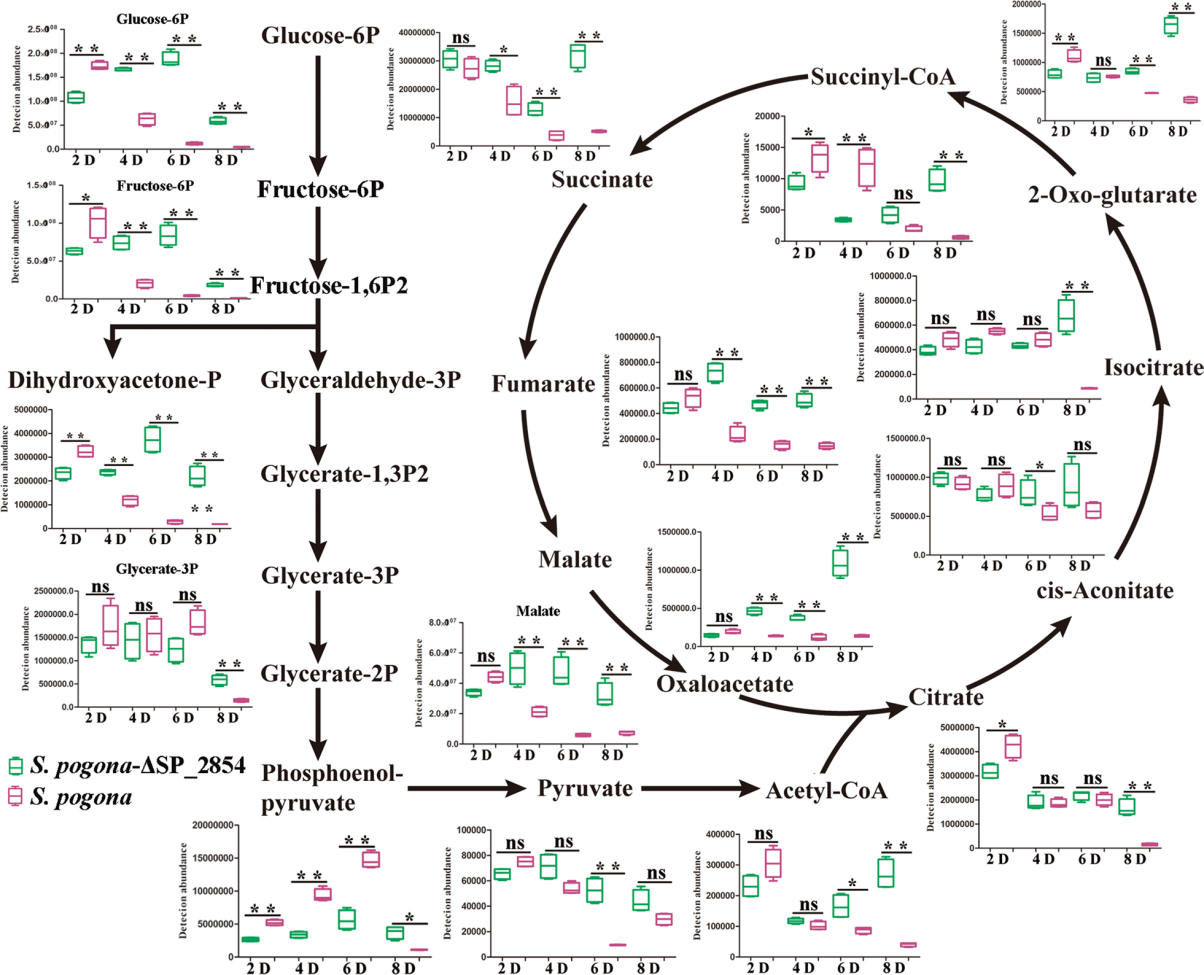
Comparison of the abundance of intracellular metabolites in the *S. pogona*-ΔSP_2854 mutant (green) and wild-type *S. pogona* (pink) related to the glycolysis pathway and TCA cycle (mean values from four biological replicates)

Butenyl-spinosyn production is not only affected by the expression level of *bus* clusters but also related to the supply capacity of precursors. Under nutrient- and oxygen-replete conditions, acetyl-CoA is predominantly derived from glucose and is the most important precursor in butenyl-spinosyn biosynthesis. Glucose determination and qRT-PCR analysis results showed that a change in SP_2854 expression could affect the glucose uptake and the expression of PTS^Glc^ genes and finally affect the glucose metabolism and the acetyl-CoA supply. To further investigate whether SP_2854 can influence butenyl-spinosyn production by regulating the supply of acetyl-CoA, we performed a targeted metabolomics analysis for specific metabolites. As a result, 27 intracellular metabolites associated with acetyl-CoA metabolism were identified and quantified by LC–MS/MS (Fig. 4, Additional file 2: DataSet S1). KEGG pathway analysis showed that these metabolites mainly participated in glycolysis and the TCA cycle. Further analysis showed that the *S. pogona*::SP_2854 mutant experienced significant increases in the abundance of several metabolites in the logarithmic phase and stationary phase, such as glucose 6-phosphate, fructose 6-phosphate, dihydroxyacetone phosphate, pyruvate, acetyl-CoA, citrate, alpha-ketoglutarate, fumarate and malate, suggesting that SP_2854 overexpression enhanced glucose metabolism and acetyl-CoA synthesis, which finally promoted butenyl-spinosyn biosynthesis (Fig. 5). For the *S. pogona*-ΔSP_2854 mutant, most metabolites involved in glycolysis and the TCA cycle, such as glucose 6-phosphate, fructose 6-phosphate, dihydroxyacetone phosphate, phosphoenolpyruvate, pyruvate, oxaloacetate, citrate, alpha-ketoglutarate and malate, were lower in abundance during the logarithmic phase compared with their abundance in the original strain, suggesting that SP_2854 deletion inhibited glucose metabolism, which was consistent with lower rates of growth and glucose consumption of the *S. pogona*-ΔSP_2854 mutant in this phase (Fig. 6). However, the abundance of these metabolites in the stationary phase or decline phase was significantly higher than their abundance in the original strain. Combined with the growth curve and glucose consumption analysis, we speculate that the *S. pogona*-ΔSP_2854 mutant still exhibited a high concentration of glucose in the extracellular environment on the 4th day of fermentation compared with that of the original strain, which enabled it to continue to use extracellular glucose for catabolism and synthesize other intermediate metabolites. Meanwhile, SP_2854 deletion caused a decrease in cellular glucose metabolism, which eventually led to the abundance of some intermediate metabolites being higher than they were in the original strain. Both mutants showed different metabolic profiles compared with the original strain, which could be due to SP_2854 being a regulatory factor that can control the expression of certain key genes that are important to enhance acetyl-CoA supply.

### Comparative proteomic analysis of the high-production mutant *S. pogona*::SP_2854 and the original strain

**Fig. 7:**
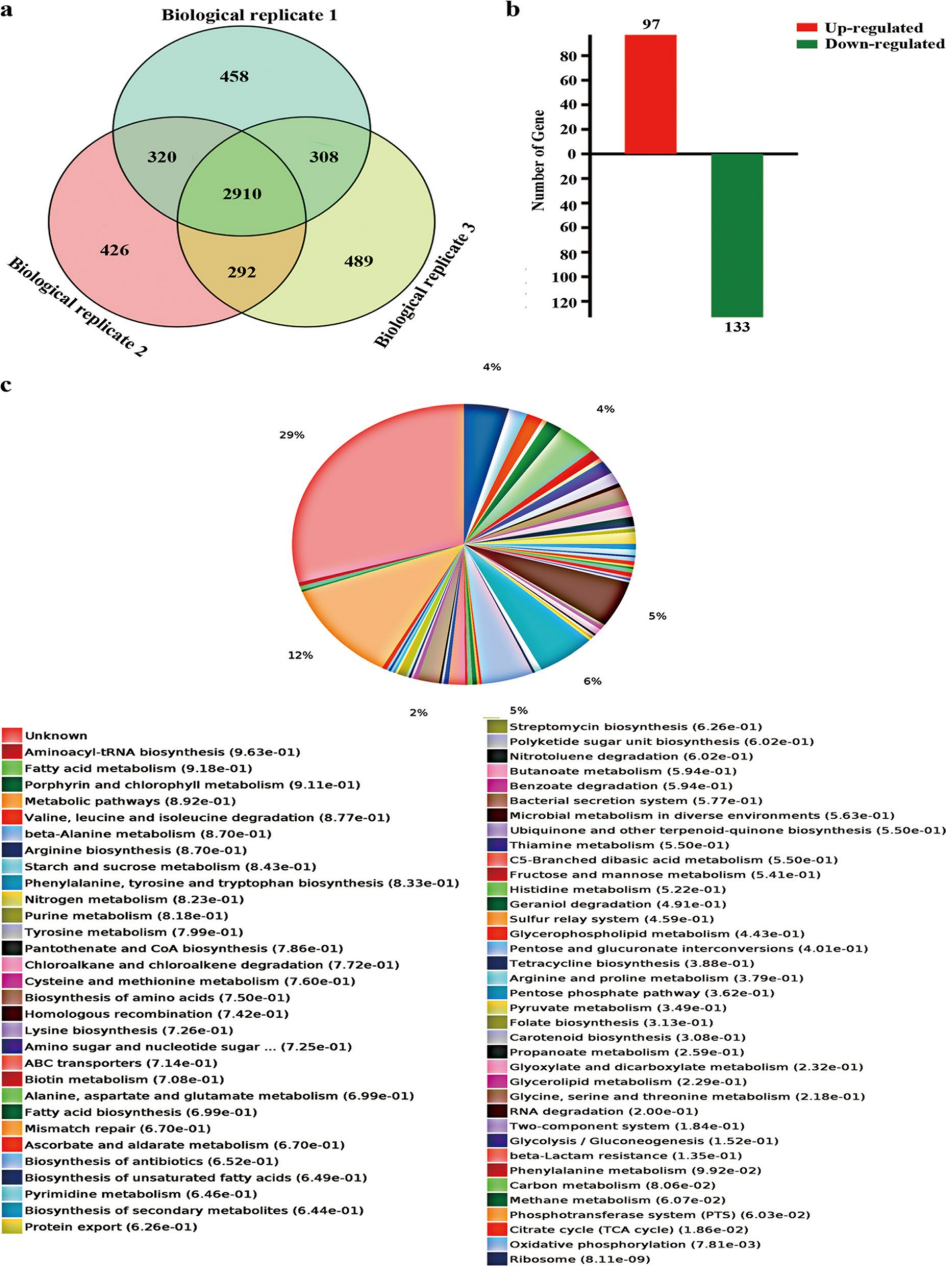
Comparative proteomic analysis of the *S. pogona*::SP_2854 mutant and wild-type *S. pogona*. **A** Venn diagram of protein identification in three biological replicates. The number of proteins is shown in each area. **B** Statistics for differentially expressed proteins. **C** KEGG pathway analysis for differentially expressed proteins

Based on morphological, physiological and biochemical characteristics, and targeted metabolome analysis, we found that SP_2854 exhibited multiple regulatory functions in the growth and development, nutrient metabolism and butenyl-spinosyn biosynthesis of *S. pogona*. In particular, SP_2854 overexpression significantly promoted butenyl-spinosyn biosynthesis. To reveal the regulatory mechanism of SP_2854, proteomic comparison between the *S. pogona*::SP_2854 mutant and the original strain was performed by using quantitative proteomics analysis based on iTRAQ labelling. It has been reported that butenyl-spinosyn is synthesized mainly in the stationary phase [31]; therefore, samples on Day 6 were collected for proteomic analysis. A total of 2910 proteins were identified and present in all biological replicates (Fig. 7a, Additional file 3: DataSet S2). A total of 230 proteins were differentially expressed (fold change > 1.33 or < 0.75, P value < 0.05), including 97 upregulated and 133 downregulated proteins, which were classified according to KEGG pathways (Fig. 7b, Additional file 4: DataSet S3). These differentially expressed proteins were involved in multiple biological processes, such as glycolysis, the pentose phosphate pathway (PP pathway), the TCA cycle, oxidative phosphorylation, amino acid metabolism, a two-component system, and ABC transporters, which suggests that SP_2854 is likely to be a global regulator (Fig. 7c).

**Fig. 8:**
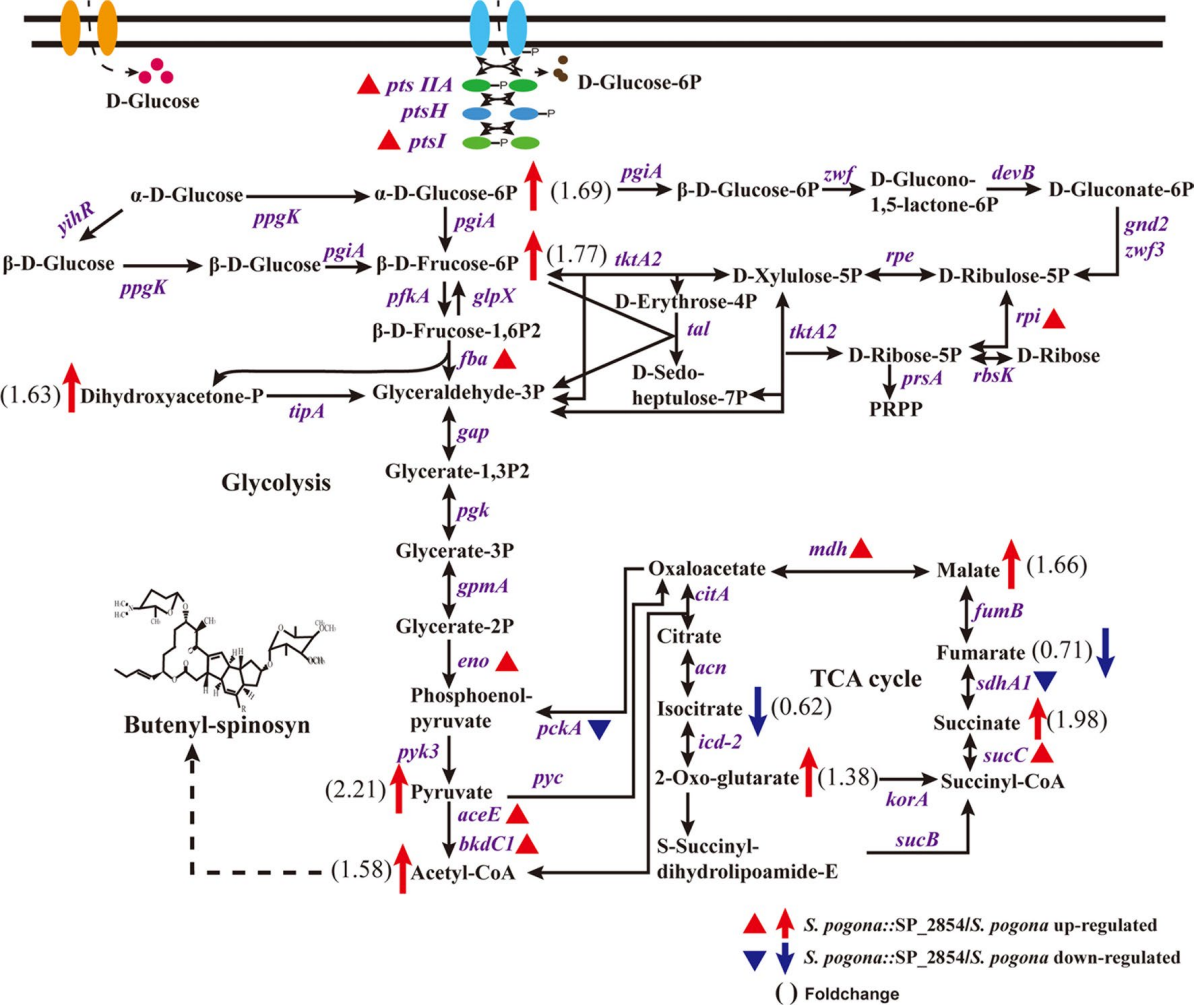
Effect of SP_2854 deletion on primary metabolism in *S. pogona*. This regulatory network is based on the analysis of differentially expressed proteins and metabolites associated with glucose transport, glycolysis, the pentose phosphate pathway and the TCA cycle. Reactions are reported according to KEGG metabolic pathway databases. A triangle represents a change in protein abundance, and an arrow represents a change in metabolite abundance

**Fig. 9:**
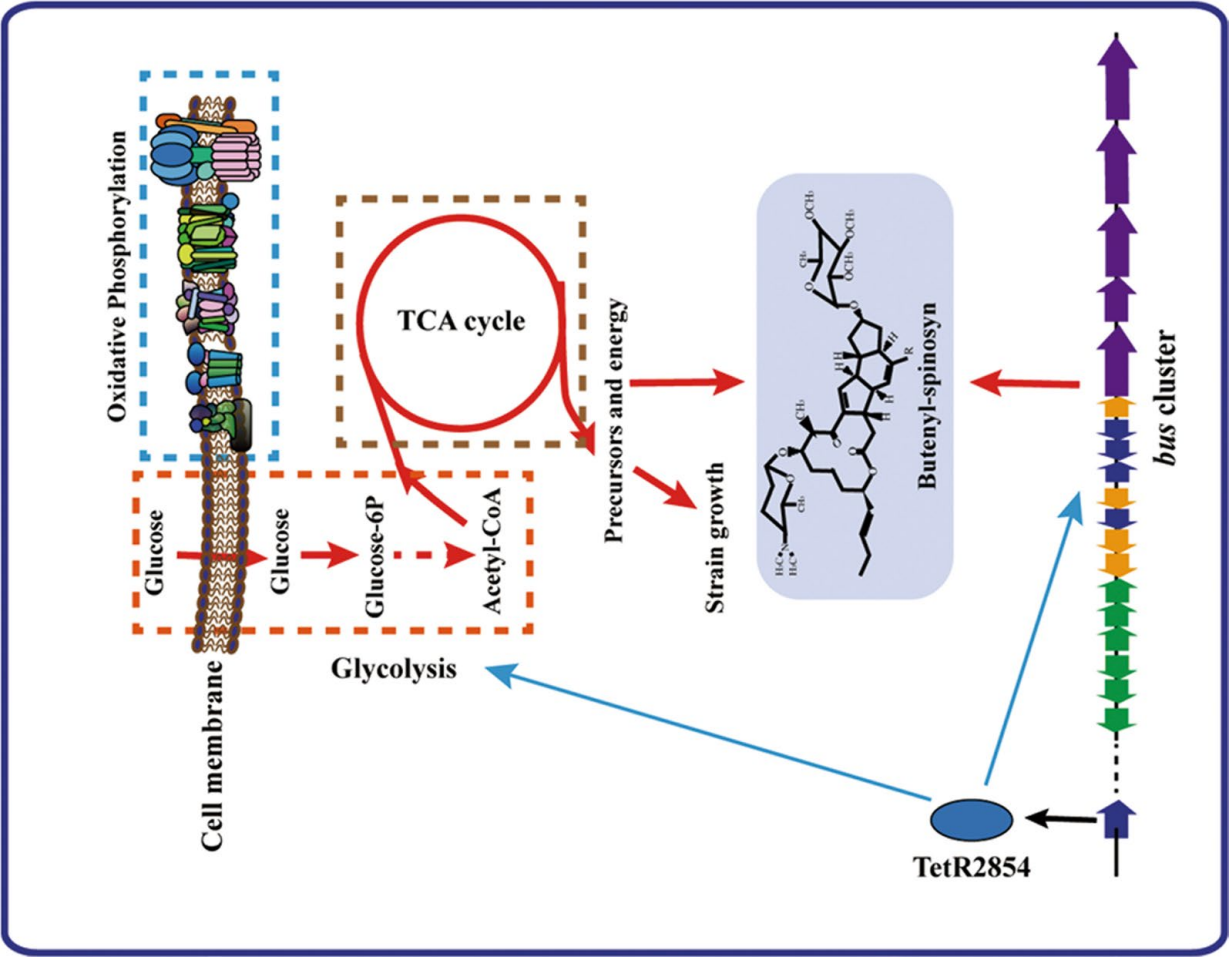
Regulatory network schematic diagram of SP_2854. The schematic diagram showed that SP_2854 can regulate the glucose metabolism and the *bus* cluster expression to promote butenyl-spinosyn biosynthesis in *S. pogona*. Red arrow: the pathway was enhanced; Cyan arrow: the regulatory effect of SP_2854

A detailed metabolic network diagram was constructed to reveal the specific metabolic differences between the *S. pogona*::SP_2854 mutant and the original strain based on an analysis of KEGG metabolic pathways (Fig. 8). We found that the abundances of PTS glucose transporter subunit IIA (*ptsIIA*), phosphoenolpyruvate phosphotransferase (*ptsI*), and their catalytic product, glucose 6-phosphate, were higher in the *S. pogona*::SP_2854 mutant than in the original strain. Glycolysis and the PP pathway are two main mechanisms whereby microorganisms metabolize glucose [33]. Throughout glycolysis, the abundance of pyruvate dehydrogenase (*aceE*, *bkdC1*) in the *S. pogona*::SP_2854 mutant was higher than that in the original strain; this enzyme is involved in the conversion of pyruvate to acetyl-CoA [34]. Other enzymes involved in glycolysis (class II fructose-bisphosphate aldolase (*fba*) and enolase (*eno*)) were also observed, and their abundance in the *S. pogona*::SP_2854 mutant was also higher than that in the original strain. Additionally, their catalytic products, such as dihydroxyacetone phosphate, phosphoenolpyruvate and acetyl-CoA, displayed high abundance in the *S. pogona*::SP_2854 mutant, indicating that SP_2854 overexpression had a significant stimulative effect on the generation of acetyl-CoA from glucose. For the PP pathway [33], however, we found no other differentially expressed proteins related to this pathway in *S. pogona*::SP_2854 except for ribose 5-phosphate isomerase (*rpi*). The acetyl-CoA produced through glycolysis will enter the TCA cycle to produce energy and several metabolic precursors for cell growth and the synthesis of other metabolites [34]. Succinyl-CoA synthetase (*sucC*) catalyses the reversible reaction of succinyl-CoA to succinate. It plays a key role as one of the catalysts involved in the TCA cycle, a central pathway in cellular metabolism [35]. Malate dehydrogenase (*mdh*) is an enzyme that reversibly catalyses the oxidation of malate to oxaloacetate using the reduction of NAD^+^ to NADH [36]. The two enzymes and the relevant metabolites, succinate, malate, oxaloacetate and NADH, were all present at higher levels under SP_2854 overexpression conditions. These data suggest that the *S. pogona*::SP_2854 mutant shows a greater metabolic capacity than the original strain in terms of glycolysis and the TCA cycle, so it can highly efficiently utilize nutrients in the extracellular environment during the analysis phase to promote butenyl-spinosyn biosynthesis in *S. pogona* (Fig. 9).

## Discussion

As a large and important family of one-component signal transduction systems, TFRs are widely associated with metabolism, antibiotic production, quorum sensing, and multidrug resistance [20–23]. Natural products, such as avermectin and calcium-dependent antibiotics, have been reported to exhibit pathway-specific regulators in their gene clusters, such as *aveR* and *cdaR* [37, 38]. Further studies have demonstrated that some TFRs directly control the expression of these genes to regulate corresponding target product biosynthesis. Unlike these natural products, no pathway-specific regulators existed in or near the *bus* cluster [2]. Therefore, the elucidation of regulatory genes with important roles in butenyl-spinosyn biosynthesis has become a research hotspot.

In this study, we characterized a TFR, SP_2854, in *S. pogona* and found that a change in its expression level can affect butenyl-spinosyn biosynthesis. The inactivation of SP_2854 decreased the production of butenyl-spinosyn by 77.3%, while its overexpression increased the production of butenyl-spinosyn by 289.7%. The differential transcription of *busA*, *busF*, *busG*, *busI*, *busP* and *busQ* in mutants *S. pogona*::SP_2854 and *S. pogona*-ΔSP_2854 further suggests that SP_2854 positively regulates butenyl-spinosyn biosynthesis. It was previously reported that some TFRs can affect other morphological, physiological and biochemical characteristics in actinomycetes [14]. In our further analysis, SP_2854 deletion also inhibited strain growth, glucose consumption and mycelial morphology. These data suggest that SP_2854 has important biological significance for maintaining normal growth and development and nutritional metabolism of *S. pogona*.

We present the first results using a targeted metabolomic platform to better understand detectable alterations in the intracellular metabolome caused by SP_2854 deletion and overexpression compared with the original strain. As a secondary metabolite, butenyl-spinosyn possesses a complex biosynthetic pathway, and its synthetic precursors include acetyl-CoA, malonyl-CoA, methylmalonyl-CoA, rhamnose, forosamine and S-adenosyl methionine [2]. As shown by the growth curve and targeted metabolome analysis, the promotion of butenyl-spinosyn by SP_2854 overexpression was due to an enhancement in cell growth and an enrichment of the precursors required. The two factors are correlated with each other since the precursors are derived from growth-related metabolic intermediates, such as acetyl-CoA, which was distributed in the complex metabolic network of *S. pogona,* as shown in Fig. 5. For example, the *S. pogona*::SP_2854 mutant showed significant increases in the abundance of several metabolites from glycolysis and the TCA cycle in the logarithmic phase and stationary phase, such as glucose 6-phosphate, fructose 6-phosphate, dihydroxyacetone phosphate, pyruvate, acetyl-CoA, citrate, alpha-ketoglutarate, fumarate and malate. For SP_2854 deletion, most metabolites involved in glycolysis and the TCA cycle in the *S. pogona*-ΔSP_2854 mutant, such as glucose 6-phosphate, fructose 6-phosphate, dihydroxyacetone phosphate, phosphoenolpyruvate, pyruvate, oxaloacetate, citrate, alpha-ketoglutarate and malate, exhibited lower abundance in the logarithmic phase compared with their abundance in the original strain. It has been demonstrated that there is a novel link between primary metabolism and butenyl-spinosyn biosynthesis, suggesting that SP_2854 is tightly linked to butenyl-spinosyn biosynthesis by regulating the primary metabolic pathway except for controlling the transcription of the *bus* cluster.

In addition, proteomic analyses were used to compare the protein expression differences between the *S. pogona*::SP_2854 mutant and original strain. A total of 97 proteins were upregulated, while 133 proteins were downregulated in the *S. pogona*::SP_2854 mutant. These differentially expressed proteins are involved in multiple biological processes, such as glycolysis, the PP pathway, the TCA cycle, oxidative phosphorylation, amino acid metabolism, a two-component system, and ABC transporters, suggesting that SP_2854 may act as a pleiotropic regulator to control butenyl-spinosyn biosynthesis. Combined with contemporaneous metabolome data, we found that most proteins and metabolites involved in primary metabolism, among which the upregulated proteins not only promote the conversion of extracellular glucose to phosphorylated glucose but also enhance glycolysis and the TCA cycle, provided sufficient precursors for other biological processes. These results may explain why the *S. pogona*::SP_2854 mutant exhibited high butenyl-spinosyn production.

## Conclusions

We have shown that SP_2854 plays a very important role in the regulation of growth and development and butenyl-spinosyn biosynthesis in *S. pogona*. Our present findings provide a strategy for the improvement of butenyl-spinosyn production based on the engineering of SP_2854, which may be applied to the augmentation of other antibiotics produced by actinomycetes that have SP_2854 homologues.

## Methods

### Bacterial strains, plasmids, and media

The bacterial strains and plasmids used in this study are listed in Table 1. The media and culture conditions used in this study are described in the Supplementary Materials and Methods.

**Table 1:** Strains and plasmids in this study

Strains/Plasmids	Description	Source
*S. pogona*		
NRRL 30,141	*S. pogona*, wild-type strain, plasmid-free parental strain	This lab
*S. pogona*-ΔSP_2854	*S. pogona*, SP_2854 deletion strain	This work
*S. pogona*::SP_2854	*S. pogona*, SP_2854 over-expressed strain	This work
*E. coli*		
DH5α	*Escherichia coli*, plasmid-free, host for general cloning	This lab
Plasmids		
pOJ260	*E. coli*-cloning vector, containing pUC18 replicon, *oriT*, Apr^R^	This lab
pKCcas9dO	*acc(3)IV, pSG5, tipA-Scocas9, j23119,* vector for expression	Huang et al. [32]
pOJ260-UA_SP_2854_-*apr*-DA_SP_2854_	pOJ260 containing homologous region flanking SP_2854, for SP_2854 deletion	This work
pKCcas9-SP_2854	pOJ260 containing *PermE* and SP_2854, for SP_2854 overexpression	This work

### SP_2854 deletion and overexpression

Targeted gene deletion mediated by homologous recombination was used to generate an SP_2854 null mutant. With *S. pogona* genomic DNA as a template, a 1.0-kb fragment upstream (UA_SP_2854_) of SP_2854 was amplified with primers F_SP_2854up_/R_SP_2854up_, and a 1.0-kb fragment downstream (DA_SP_2854_) of SP_2854 was amplified with primers F_SP_2854down_/R_SP_2854down_. With pOJ260 plasmid DNA as template, a 1.3-kb apramycin resistance gene (*apr*) was amplified with primers F_*apr*_/R_*apr*_. The 5′-ends of R_SP_2854up_ and F_SP_2854down_ have a 35-nt overlapping sequence, allowing the joining of UA_SP_2854_, *apr* and DA_SP_2854_ in a subsequent fusion PCR. The resulting 3.3 kb fusion fragment was digested with *Hin*d III and *Eco*R I and cloned into the corresponding restriction sites of pOJ260, yielding pOJ260-UA_SP_2854_-*apr*-DA_SP_2854_. Then, this plasmid was transformed into *S. pogona* by protoplast transformation [39]. Transformants were selected directly on R5 plates containing 50 µg/mL apramycin. Since pOJ-260 is a suicide plasmid, it cannot be replicated in *S. pogona* and will eventually be lost as the strain grows. Therefore, the chromosome structure at the SP_2854 locus of several Apra^R^ colonies was analysed by PCR using primers F_SP_2854up_/R_*apr*_ and by sequencing (Sangon Biotech, Shanghai), and the mutant that was successfully verified was named *S. pogona*-ΔSP_2854.

For SP_2854 overexpression, plasmid pKCcas9dO with the synthetic promoter J23119 was selected as the editing tool [32]. *S. pogona* NRRL 30141 was used as the template for SP_2854 amplification by PCR, and the primers were F_SP_2854_/R_SP_2854_. The resulting 703-bp fragment was digested with *Spe* I and *Hin*d III and cloned into the corresponding restriction sites of pKCcas9dO, yielding pKCcas9-SP_2854. Then, this plasmid was transformed into *S. pogona* by protoplast transformation. Transformants were selected directly on R5 plates containing 50 µg/mL apramycin. The chromosome structure of several Apra^R^ colonies was analysed by PCR using primers F_*apr*_/R_*apr*_, and the mutant that was successfully verified was named *S. pogona*::SP_2854.

### Cultivation profile analysis of the *S. pogona*-ΔSP_2854 and *S. pogona*::SP_2854 mutants

The cells were collected in SFM at 8th day to determine the butenyl-spinosyn production by using an HPLC system (1290, Agilent, USA). The sample processing and testing methods as described previously [9]. The samples were cultured with ethyl acetate at a 1:1 volumetric ratio, incubated for 48 h, and then centrifuged at 9000×*g* for 15 min. The supernatants were filtered through 0.22 µm Millipore filters. After filtration, a 20 µL aliquot of each supernatant was loaded onto a C18 column (AQ12S05-1546WT, YMC, Kyoto, Japan) and eluted with elution buffer (buffer A: 10% (v/v) acetonitrile, buffer B: 90% (v/v) acetonitrile) at 1.0 mL/min. The following gradient of buffer B was applied: 0 min, 0%; 2 min, 0%; 20 min, 100%; 22 min, 100%; 23 min, 0%; and 25 min, 0%. The detection wavelength was set to 250 nm during the analysis. Spinosyn 6-methyl β, which was previously purified and identified in our laboratory, was used as an internal standard because it fulfilled the requirements in terms of chemical similarity to the analyte.

An optical density of 600 nm (OD_600_) was used to determine the cell concentration. Cells were collected every 12 h during fermentation for growth curve measurements by UV scanning, and a certain dilution ratio was chosen to ensure that the measured value ranged from 0.2 to 0.8. Supernatants were collected every 12 h during fermentation to determine the glucose concentration by using a glucose assay kit (Sangon Biotech, Shanghai) until the glucose was fully consumed.

For observations of mycelium morphology, cells were collected on the 2nd, 4th and 6th days and soaked with 2.5% glutaraldehyde at 4 °C overnight and then subjected to gradient dehydration using ethanol solutions (30%, 50%, 70%, 90%, and 100%). The samples were dried naturally, sprayed with platinum, and subsequently observed by scanning electron microscopy (Hitachi SU8010, Japan). For sporulation, equal strains were streaked on CSM, BHI and TSM solid media and incubated at 30 °C for observation of sporulation for 5 days.

All of the experiments were performed in triplicate.

### Determination of intracellular metabolites associated with energy metabolism by LC–MS/MS

For targeted metabolomic analysis, samples were collected on Day 2, Day 4, Day 6 and Day 8, and the sample treatment methods used were as described by Yang et al. with some modifications [40]. Ten millilitres of culture broth was collected and centrifuged, and the samples were transferred to EP tubes and mixed with 400 μL of methanol/acetonitrile (1:1, v/v). The tubes were vortexed for 30 s, incubated for 10 min at − 20 °C, and then centrifuged at 14,000*g* for 15 min at 4 °C. The supernatants were collected and dried with nitrogen, and the lyophilized powder was stored at − 80 °C prior to analysis. Targeted metabolite analysis was performed using an LC–MS/MS system. The dried metabolites were dissolved in 100 μL of acetonitrile/H_2_O (1:1, v/v) and centrifuged (13,000*g*) for 15 min. Electrospray ionization was conducted with an Agilent 1290 Infinity chromatography system and AB Sciex QTRAP 5500 mass spectrometer. NH_4_COOH (15 mM) and acetonitrile were used as mobile phases A and B, respectively. A binary solvent gradient was used as follows: A, NH_4_COOH; B, 0–18 min at 90% to 40% acetonitrile; 18–18.1 min at 40% to 90% acetonitrile; and 18.1–23 min at 90% acetonitrile. The LC–MS/MS was operated in negative mode under the following conditions: source temperature, 450 °C; ion source gas 1, 45; ion source gas 2, 45; curtain gas, 30; and ion spray voltage floating (ISVF), − 4500 V. All of the samples were performed in quadruplicate.

### Extraction, preparation and LC–MS/MS analysis of whole proteins

The cells of the *S. pogona*-ΔSP_2854 mutant and the original strain were harvested (centrifugation at 9000*g* for 10 min at 4 °C) after 6 days of culture, washed four times by resuspending the cell pellet in 20 mL fresh PBS (10 mM, pH 7.8, prechilled at 4 °C), and quickly frozen in liquid nitrogen (three independent repeats). Protein extraction and 2D LC–MS/MS analysis were performed as described previously with slight modifications [41]. Detailed procedures are described in the Additional file 1.

LC–MS/MS data were analysed for protein identification and quantification using ProteinPilot software v.4.5 (Sciex Inc., USA). The utilized protein database was the protein sequence set of all *Saccharopolyspora* strains. The false discovery rate (FDR) was estimated via a reverse database strategy, and only proteins below the threshold of 1% of FDR were further considered. The differentially abundant proteins (DAPs) were defined in the iTraq experiment by the following criteria: unique peptides ≥ 1, P value < 0.05, and fold change > 1.33 or < 0.75. Only proteins present in all replicate samples were considered for subsequent analysis. The metabolic pathway analysis of DAPs was conducted using the KEGG database (http://www.genome.jp/kegg/).

### RNA isolation, cDNA synthesis, and quantitative reverse transcription PCR (qRT–PCR)

The total RNAs of the WT strain and mutants were separately isolated by using TRIzol Reagent (Sangon Biotech, Shanghai). cDNA synthesis was performed by using a High-Capacity cDNA Archive Kit (Fermentas) in accordance with the manufacturer’s instructions. Real-time qPCR amplification was performed using Power SYBRR Green PCR Master Mix (Applied Biosystems) as previously described [9]. The primer sequences used in qRT–PCR were designed with Primer Premier 5.0 and are listed in Additional file 1: Table S2. The transcript generated from the 16S rRNA gene was used for normalization. The results are expressed as the means from three replicate experiments.

### Statistical analysis

All data in this study were stated as means ± standard error of mean (SEM), and analysis by Student’t t-test, with *p < 0.05, **p < 0.01, ns, no significant.

## Supplementary Information

**Additional file 1: Table S1.** Oligonucleotides used in this study. **Table S2.** qRT-PCR primers used in this study. **Figure S1.** Mass spectrum identification of butenyl-spinosyn. (A) Chromatographic peak detection spectrum in 13.0 min. (B) Chromatographic peak detection spectrum of secondary spectrum in 13.0 min. MS identification results showed that (M + H) ^+^ ions at m/z = 633.17 (black arrow) contained a rhamnose ion fragment of 189 molecular mass, which was confirmed as a butenyl-spinosyn component spinosyn 6-methyl β. **Figure S2.** Sporulation observation of the *S. pogona*-ΔSP_2854 (1), *S. pogona*::SP_2854 (2), and *S. pogona* (3) in the BHI, CSM and TSB solid media. The strains were grown on the BHI, CSM and TSB solid media and photographed on days 5.

**Additional file 2: DataSet S1.** Transcriptomic data of mutants *S. pogona*-ΔSP_2854 and *S. pogona*::SP_2854.

**Additional file 3: DataSet S2.** Comparative proteomic analysis of the *S. pogona*::SP_2854 mutant and *S. pogona* at 6th day.

**Additional file 4: DataSet S3.** Differentially expressed proteins analysis of the S. pogona::SP_2854 mutant and wild-type S. pogona at 6th day.

## Data Availability

All data generated or analyzed during this study are included in this published article and its Additional files 1, 2, 3 and 4.

## Abbreviations

CDA: Calcium-dependent antibiotic
TFRs: TetR family regulatory factors
qRT–PCR: Quantitative reverse transcription PCR
SEM: Scanning electronic microscopy
LC–MS/MS: Liquid chromatography-tandem mass spectrometry
KEGG: Kyoto encyclopedia of genes and genomes
TCA: Tricarboxylic acid
iTRAQ: Isobaric tags for relative and absolute quantification
ABC: ATP -binding cassette
PTS: Phosphotransferase system
PP: Pentose phosphate
*apr*: Apramycin resistance gene
HPLC: High performance liquid chromatography
SFM: Synthetic fermentation medium
CSM: Complete synthetic medium
FDR: False discovery rate
DAPs: Differentially abundant proteins
