# Supplementary material for: A TetR family transcriptional regulator, SP_2854 can affect the butenyl-spinosyn biosynthesis by regulating glucose metabolism in Saccharopolyspora pogona
Source: Microb Cell Fact. 2022 May 14;21:83. doi: 10.1186/s12934-022-01808-2 (PMC9107242; doi:10.1186/s12934-022-01808-2)
Supplement: Supplementary file 1 — Additional file 1: Table S1. Oligonucleotides used in this study. Table S2. qRT-PCR primers used in this study. Figure S1. Mass spectrum identification of butenyl-spinosyn. (A) Chromatographic peak detection spectrum in 13.0 min. (B) Chromatographic peak detection spectrum of secondary spectrum in 13.0 min. MS identification results showed that (M + H) + ions at m/z = 633.17 (black arrow) contained a rhamnose ion fragment of 189 molecular mass, which was confirmed as a butenyl-spinosyn component spinosyn 6-methyl β. Figure S2. Sporulation observation of the S. pogona-ΔSP_2854 (1), S. pogona::SP_2854 (2), and S. pogona (3) in the BHI, CSM and TSB solid media. The strains were grown on the BHI, CSM and TSB solid media and photographed on days 5. [file 12934_2022_1808_MOESM1_ESM.doc]

**Supporting Information**

**A TetR family transcriptional regulator, SP_2854 can affect the butenyl-spinosyn biosynthesis by regulating glucose metabolism in *Saccharopolyspora pogona***

Jie Rang^1,2#^, Ziyuan Xia^1#^, Ling Shuai^1^, Li Cao^1^, Yang Liu^1^, Xiaomin Li^1^, Jiao Xie^1^, Yunlong Li^1^, Shengbiao Hu, Qingji Xie^2^*, and Liqiu Xia^1^*

**^1^**Hunan Provincial Key Laboratory for Microbial Molecular Biology, State Key Laboratory of Development Biology of Freshwater Fish, College of Life Science, Hunan Normal University, 410081 Changsha, China

^2^Key Laboratory of Chemical Biology and Traditional Chinese Medicine Research (MOE of China), National & Local Joint Engineering Laboratory for New Petro-chemical Materials and Fine Utilization of Resources, College of Chemistry and Chemical Engineering, Hunan Normal University, Changsha 410081, China

#These authors contributed equally to this work.

*Corresponding Author’s E-mail: [xieqj@hunnu.edu.cn](mailto:xieqj@hunnu.edu.cn) (Qingji Xie); [xialq@hunnu.edu.cn (Liqiu Xia).](mailto:xialq@hunnu.edu.cn;)

**Supplementary Materials and Methods**

**Bacterial strains, plasmids, and media**

*Escherichia coli* (*E. coli*) DH5α was used to construct the recombinant plasmids. *E. coli* BL21 was used as the host for heterologous protein production. All *E. coli* strains were grown at 37 °C in Luria-Bertani broth. The complete synthetic medium (CSM; per liter: 10 g of glucose, 45 g of trypticase soy broth, 9 g of yeast extract, and 2.2 g of MgSO_4_·7H_2_O) was used for *S. pogona* and its derivatives activation. For butenyl-spinosyn fermentation and other phenotypic analysis, synthetic fermentation medium (SFM; per liter: 1 g of KNO_3_, 0.01 g of FeSO_4_·7H_2_O, 0.5 g of K_2_HPO_4_·3H_2_O, 0.5 g of MgSO_4_·7H_2_O, 20 g of glucose, 4 g of yeast extract, 4 g of tryptone; pH of 7.2) was employed. SFM with different concentrations of K_2_HPO_4_·3H_2_O (0, 2.5 × 10^-3^ and 4 μm) were used to analyse the effects of phosphate on the growth development and butenyl-spinosyn biosynthesis of *S. pogona*. The R5 medium (per liter: 103 g of sucrose, 10 g of glucose, 0.1 g of casein hydrolysate, 5 g of yeast extract, 5.73 g of TES, 0.25 g of K_2_SO_4_, 10.12 g of MgCl_2_·6H_2_O, 2 mL of microelement solution, and 15 g of agar) was used for *S. pogona* protoplast transformation. R5 medium (100 mL) was supplemented with 1 mL KH_2_PO_4_ (0.5%), 10 mL CaCl_2_·2H_2_O (3.68%), and 10 mL TES buffer (5.73%, pH7.2) before use. If necessary, 50 µg/ml apramycin (Apra) was added into the above media.

**Whole proteins extraction, preparation and LC-MS/MS analysis of the whole proteins**

Each protein extract (300 µg) was reduced with 500 mM dithiothreitol (DTT) at room temperature for 60 min and then alkylated with 500 mM iodoacetamide at room temperature in the dark for 60 min. Excess iodoacetamide was quenched with 15 mM DTT for 15 min at room temperature. The sample solutions were then incubated overnight with trypsin at a trypsin/protein ratio of 1:50 (w/w) at 37 °C. Tryptic peptides were desalted and concentrated on an Oasis HLB sample cartridge column (Waters Corporation, MA, USA). Subsequently, the samples were labeled with an iTraq reagent in accordance with the manufacturer’s protocol (ABSciex, Framingham, MA, USA) and then lyophilized for further 2D online LC-MS analysis.

2D chromatography was conducted on an Eksigent nano LC-Ultra™1D System (AB SCIEX, Concord, ON). The lyophilized SCX fractions were re-dissolved in 2% acetonitrile and 0.1% formic acid and then loaded on a ChromXP C18 (3 µm, 120 Å) nanoLC trap column. Online trapping and the desalting were performed at 2 µL/min for 10 min with 100% solvent A. The solvents were composed of water/acetonitrile/formic acid (A, 98%/2%/0.1%; B, 2%/98%/0.1%). An elution gradient of 5%–38% acetonitrile (0.1% formic acid) in 70 min gradient was then employed on an analytical column (75 µm × 15 cm C18, 3 μm, 120 Å, ChromXP Eksigent). LC-MS/MS analysis was performed using a TripleTOF 5600 System (SCIEX, Concord, ON) fitted with a Nanospray III source. Data were acquired at an ion spray voltage of 2.4 kV, a curtain gas of 30 PSI, a nebulizer gas of 5 PSI, and an interface heater temperature of 150 °C. MS was operated with TOF-MS scans. For information dependent acquisition (IDA), the survey scans were acquired in 250 ms, and up to 30 product ion scans (80 ms) were collected if the threshold of 260 counts was exceeded and with a +2 to +5 charge state. Rolling collision energy setting was applied to all precursor ions for collision-induced dissociation. Dynamic exclusion was set to 16 s.

**Table S1. Oligonucleotides used in this study**

| **Primers** | **Sequences (5'-3')** | **Source/reference** |
| --- | --- | --- |
| FSP_2854up | GCT**AAGCTT**GTTTTGGAACCCGACAGGAT | This work |
| RSP_2854up | TCCTTTTTTTCTGCGCGTAATCTGCTGCTTGCAAACGAAATCACCGTCATGACAA | This work |
| FSP_2854down | TATTTAAAGATCCACTAGTATGCAGGTCGACGGATGCCTCGGACCTAAAGATACT | This work |
| RSP_2854down | ACG**GGATCC**CGAGTTGTTCACCGATCATC | This work |
| F*_apr_* | TTTGCAAGCAGCAGATTACG | This work |
| R*_apr_* | ATCCGTCGACCTGCATACTA | This work |
| FSP_2854 | TTGG**ACTAGT**TCTTTAGGTCCGAGGCACAG | This work |
| RSP_2854 | GTC**AAGCTT**TGTCATGACGGTGATTTCGC | This work |

Note: reatricition enzyme sites were bold; overlaping sequences were underlined.

**Table S2 qRT-PCR primers used in this study**

| **Gene ID** | **Name** | **Sequence (5'-3')** |
| --- | --- | --- |
| SP_3547 | F*_busA_* | GCAACCTCCCTGGATTACGG |
|  | R*_busA_* | ATGAACACGCCGTATCCACC |
| SP_3548 | F*_busF_* | ACCAGGTGGACTTCTCGTGC |
|  | R*_busF_* | ATCCCGCTGCCTATTTCTCG |
| SP_3549 | F*_busG_* | TCCCGCTCAACCTGTTCCTG |
|  | R*_busG_* | CTGCTCATCCGGCAAGCAGA |
| SP_3551 | F*_busI_* | GTCCTTCCATGCCCTGTTTC |
|  | R*_busI_* | AGGCCGTCGATCAGTTCTTT |
| SP_3558 | F*_busP_* | TGCGACTGCCTGTGGACTTG |
|  | R*_busP_* | TGCCTGTTCCTGGGCTTCTC |
| SP_3559 | F*_busQ_* | CCCCACGACCATCAATCCAG |
|  | R*_busQ_* | GATTTCGTCAGCGGCAAAGG |
| *ptsI* | F*_ptsI_* | CCACGGCCTGCTGTTTCTC |
|  | R*_ptsI_* | TCGTCTTCCAGTCCGGTGAAT |
| *ptsH* | F*_ptsH_* | GACACTGATCCGGTGGAGGC |
|  | R*_ptsH_* | TGTTGTCGAGGTCCGAGGC |
| *ptsIIA* | F*_ptsIIA_* | CTGGGCATCGACACGGTGAA |
|  | R*_ptsIIA_* | CCTCGCGGACATCGGTCA |
| *ptsIIBC* | F*_ptsIIBC_* | GAGCCGCTGGAGTTCTCGTT |
|  | R*_ptsIIBC_* | GATGCCGAAGTTGAGCACGT |





**Figure S1. Mass spectrum identification of butenyl-spinosyn.** (A) Chromatographic peak detection spectrum in 13.0 min. (B) Chromatographic peak detection spectrum of secondary spectrum in 13.0 min. MS identification results showed that (M + H) ^+^ ions at m/z = 633.17 (black arrow) contained a rhamnose ion fragment of 189 molecular mass, which was confirmed as a butenyl-spinosyn component spinosyn 6-methyl β.

**

**

**Figure S2. Sporulation observation of the *S. pogona*-ΔSP_2854 (1), *S. pogona*::SP_2854 (2), and *S. pogona* (3) in the BHI, CSM and TSB solid media.** The strains were grown on the BHI, CSM and TSB solid media and photographed on days 5.
